# Supplementary material for: Identification of Potential Muscle Biomarkers in McArdle Disease: Insights from Muscle Proteome Analysis
Source: Int J Mol Sci. 2022 Apr 22;23(9):4650. doi: 10.3390/ijms23094650 (PMC9100117; doi:10.3390/ijms23094650)
Supplement: Supplementary file 1 [file ijms-23-04650-s001.zip › ijms-1553324_File 1_Suppl_Figure S1.pdf]

Identification of Potential Muscle Biomarkers in McArdle Disease: Insights from Muscle Proteome Analysis

Inés García-Consuegra [1,2,†], Sara Asensio-Peña [1,†], Rocío Garrido-Moraga [1], Tomàs Pinós [2,3], Cristina Domínguez-González [1,2], Alfredo Santalla [4], Gisela Nogales-Gadea [5], Pablo Serrano-Lorenzo [1,2], Antoni L. Andreu [6], Joaquín Arenas [1,2], José L. Zugaza [7,8], Alejandro Lucia [1,9] and Miguel A. Martín [1,2,*]

[1] Mitochondrial and Neuromuscular Disorders Group, Hospital 12 de Octubre Health Research Institute (imas12), 28041 Madrid, Spain; inesgcg@hotmail.com (I.G.-C.); sarita.asensio@gmail.com (S.A.-P.); rociogarridorgm@gmail.com (R.G.-M.); cdgonzalez@salud.madrid.org (C.D.-G.); pserranolorenzo.imas12@h12o.es (P.S.-L.); joaquin.arenas@salud.madrid.org (J.A.); alejandro.lucia@universidadeuropea.es (A.L.)
[2] Centro de Investigación Biomédica en Red de Enfermedades Raras (CIBERER), 28029 Madrid, Spain; tomas.pinos@vhir.org
[3] Mitochondrial and Neuromuscular Disorders Unit, Vall d'Hebron Institut de Recerca, Universitat Autònoma de Barcelona, 08193 Barcelona, Spain
[4] Department of Computer and Sport Sciences, Universidad Pablo de Olavide, 41013 Sevilla, Spain; asanher@upo.es
[5] Grup de Recerca en Malalties Neuromusculars i Neuropediàtriques, Department of Neurosciences, Institut d'Investigacio en Ciencies de la Salut Germans Trias i Pujol i Campus Can Ruti, Universitat Autònoma de Barcelona, 08916 Barcelona, Spain; gnogales@igtp.cat
[6] EATRIS, European Infrastructure for Translational Medicine, 1019 Amsterdam, The Netherlands; toniandreu@eatris.eu
[7] Achucarro Basque Center for Neuroscience, Science Park of the UPV/EHU, and Department of Genetics, Physical Anthropology, and Animal Physiology, Faculty of Science and Technology, UPV/EHU, 48940 Leioa, Spain; joseluis.zugaza@ehu.es
[8] IKERBASQUE, Basque Foundation for Science, Plaza Euskadi 5, 48009 Bilbao, Spain
[9] Faculty of Sport Sciences, Universidad Europea de Madrid, 28670 Madrid, Spain
* Correspondence: mamcasanueva.imas12@h12o.es
† These authors equally contributed.

Supplementary Figure S1.

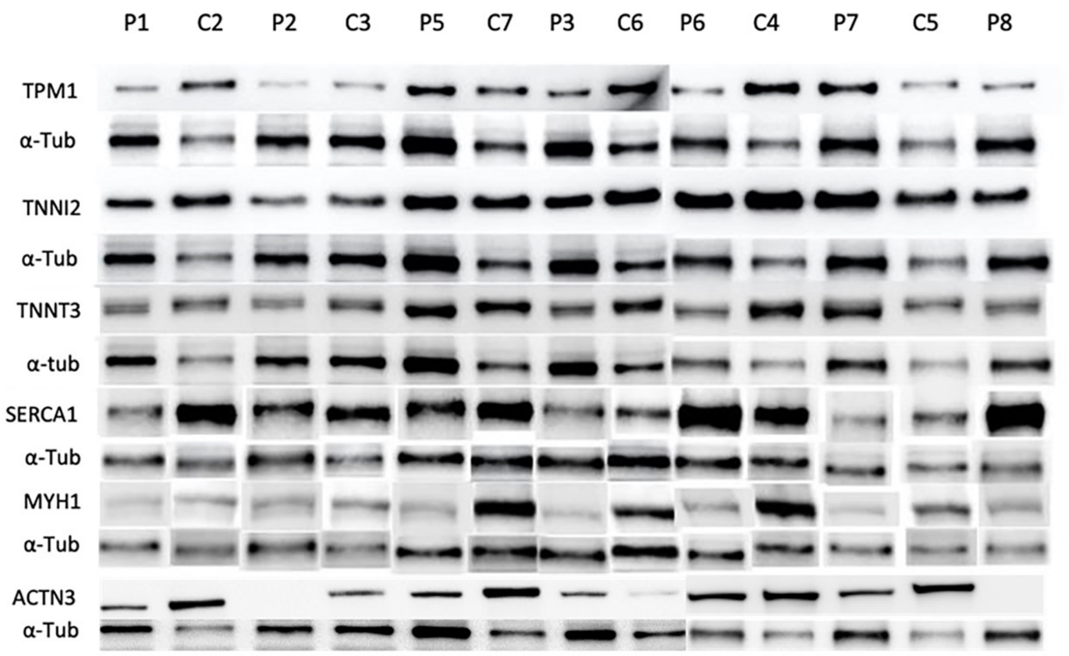

Supplementary Figure S1. Western-blot analysis in skeletal muscle tissue of the candidate proteins that were classified in the 'very strong' and 'strong' categories in the 'molecular characterization' of GSDV by means of artificial neural network analysis. Blots from all GSDV patients and controls (see tables 1 and 2 in the article main text; and Figure 2 for protein levels analysis). alpha-tubulin was used as loading control. P, patients. C, controls. Abbreviations: ACTN3, alpha-actinin-3; ATP2A1, sarcoplasmic/endoplasmic reticulum calcium ATPase 1 (also abbreviated as SERCA1); MYH1, myosin 1; TNNI2, troponin I2, fast skeletal type; TNNT3, troponin T3, fast skeletal type; TPM1, tropomyosin alpha-1 chain.
