# Supplementary material for: Identification of Potential Muscle Biomarkers in McArdle Disease: Insights from Muscle Proteome Analysis
Source: Int J Mol Sci. 2022 Apr 22;23(9):4650. doi: 10.3390/ijms23094650 (PMC9100117; doi:10.3390/ijms23094650)
Supplement: Supplementary file 1 [file ijms-23-04650-s001.zip › ijms-1553324_File 3_Suppl Tables_S2-S4_Legend_Table S1.pdf]

Identification of Potential Muscle Biomarkers in McArdle Disease: Insights from Muscle Proteome Analysis

Inés García-Consuegra [1,2,†], Sara Asensio-Peña [1,†], Rocío Garrido-Moraga [1], Tomàs Pinós [2,3], Cristina Domínguez-González [1,2], Alfredo Santalla [4], Gisela Nogales-Gadea [5], Pablo Serrano-Lorenzo [1,2], Antoni L. Andreu [6], Joaquín Arenas [1,2], José L. Zugaza [7,8], Alejandro Lucia [1,9] and Miguel A. Martín [1,2,*]

[1] Mitochondrial and Neuromuscular Disorders Group, Hospital 12 de Octubre Health Research Institute (imas12), 28041 Madrid, Spain; inesgcg@hotmail.com (I.G.-C.); sarita.asensio@gmail.com (S.A.-P.); rociogarridorgm@gmail.com (R.G.-M.); cdgonzalez@salud.madrid.org (C.D.-G.); pserranolorenzo.imas12@h12o.es (P.S.-L.); joaquin.arenas@salud.madrid.org (J.A.); alejandro.lucia@universidadeuropea.es (A.L.)
[2] Centro de Investigación Biomédica en Red de Enfermedades Raras (CIBERER), 28029 Madrid, Spain; tomas.pinos@vhir.org
[3] Mitochondrial and Neuromuscular Disorders Unit, Vall d'Hebron Institut de Recerca, Universitat Autònoma de Barcelona, 08193 Barcelona, Spain
[4] Department of Computer and Sport Sciences, Universidad Pablo de Olavide, 41013 Sevilla, Spain; asanher@upo.es
[5] Grup de Recerca en Malalties Neuromusculars i Neuropediàtriques, Department of Neurosciences, Institut d'Investigacio en Ciencies de la Salut Germans Trias i Pujol i Campus Can Ruti, Universitat Autònoma de Barcelona, 08916 Barcelona, Spain; gnogales@igtp.cat
[6] EATRIS, European Infrastructure for Translational Medicine, 1019 Amsterdam, The Netherlands; toniandreu@eatris.eu
[7] Achucarro Basque Center for Neuroscience, Science Park of the UPV/EHU, and Department of Genetics, Physical Anthropology, and Animal Physiology, Faculty of Science and Technology, UPV/EHU, 48940 Leioa, Spain; joseluis.zugaza@ehu.es
[8] IKERBASQUE, Basque Foundation for Science, Plaza Euskadi 5, 48009 Bilbao, Spain
[9] Faculty of Sport Sciences, Universidad Europea de Madrid, 28670 Madrid, Spain
* Correspondence: mamcasanueva.imas12@h12o.es
† These authors equally contributed.

Supplementary Table S1 (Supplementary Ms_Excel Table S1) legend: Proteins identified by quantitative proteomic analysis of skeletal muscle biopsies from 8 GSDV patients and 8 controls using isobaric tags for relative and absolute quantitation (iTRAQ) labelling and reversed-phase liquid chromatography mass spectrometry (RP-LC-MS/MS)

Characteristics of the 178 proteins identified by iTRAQ- and RP-LC-MS/MS. Double parallel labeling was performed using reagents 113 y 115 for patients' pools and reagents 114 and 116 for controls pools. Numerical values are displayed for each individual labeling (pools 113 to 116) referenced to the values for the 113 patients' pool. Values for proteins showing two-fold more expression at least in one control pool than in 113 patients' pool are displayed in bold and are blue colored.

Supplementary Table S2. GSDV-associated processes and number of the proteins associated to each one, after GSDV characterization by applying a strategy of systems biology.

**Motive**	**Level**	**Submotive**	**Number of proteins in the submotive**	**Number of proteins in the motive**
Glycogenolysis blockade	C	-	1	1
Downregulation of muscle sodium/potassium pumps	S	-	8	8
Elevated cytosolic calcium levels	S	-	1	40
		Persistent contraction of muscle fibers	21	
		Activation of phospholipase A2 and proteases	5	
		Calcium mitochondrial function modulation	13	
Oxidative stress	S	Producing oxidative stress	7	9
		Counteracting oxidative stress	2	
Modulation of alternative metabolic pathways for energy obtainment	S	Increased glucose uptake	10	14
		Increased lactate uptake	1	
		Enhanced fat metabolism	1	
		Creatine kinase shuttle	2	
		Total	72	

GSDV disease was characterized as causative motive (mutations in PYGM) and symptomatic motives (processes that occur in the skeletal muscle due to ATP deficiency and ADP and Pi accumulation). C: causative; S: symptomatic process.

Supplementary Table S3. Relationship between protein candidates and GSDV motives and submotives as described by molecular characterization.

**Gene Name**	**UniProt Code**	**Downregulation of muscle sodium/potassium pumps**	**Elevated cytosolic calcium levels**	*Persistent contraction of muscle fiber*	*Activation of phospholipase A2 and proteases*	*Calcium mitochondrial function modulation*	**Oxidative stress**	*Producing oxidative stress*	*Counteracting oxidative stress*	**Modulation of alternative metabolic pathways for energy obtainment**	*Increased glucose uptake*	*Creatine kinase shuttle*
MYH1	**P12882**	18	91	90	15	17	7	9	15	14	15	34
ATP2A1	O14983	11	90	7	14	7	20	18	15	7	10	15
TPM1	**P09493**	5	92	71	7	5	5	6	17	17	13	14
TNNI2	P48788	18	93	79	15	5	16	15	15	16	19	14
TNNT3	**P45378**	15	95	78	15	13	13	15	14	19	15	15
PYGM	**P11217**	16	26	10	7	16	13	15	14	10	13	16
PDLIM7	Q9NR12	5	31	66	15	6	14	15	14	6	6	14
ACTN3	**Q08043**	16	56	53	6	6	16	16	14	5	6	14
MYBPC2	**Q14324**	16	60	55	11	6	16	16	14	15	13	14
FHL3	**Q13643**	16	38	25	7	5	6	6	14	15	15	14
FHL1	**Q13642**	16	48	39	7	16	7	9	17	17	15	14
MYLPF	**Q96A32**	34	53	16	16	15	15	9	15	12	14	34
AKR1B1	P15121	14	26	10	6	13	15	14	14	15	15	14
CMBL	Q96DG6	15	38	10	15	17	15	15	34	19	19	34

Motives (bold) and submotives (italicized) related to suppl. Table 3. Numbers indicate the artificial neural network scores. Bold entries are proteins showing at least two-fold higher expression in one control than in patient 113 (see methods section in the article).

Supplementary Table S4. Categorization of the ranking score obtained after evaluation of candidate genes and GDSV according to its molecular characterization by means of artificial neural networks (ANNs).

ANN Score	ANN Category	p-value
>91	Very strong	<0.01
76-91	Strong	0.01 to 0.05
40-76	Medium-strong	0.05 to 0.25
<40	Weak	>0.25

ANN: artificial neural network. ANNs evaluate possible relations among protein sets or regions inside the network providing a predictive score that quantifies the probability of the existence of a relationship between the evaluated regions. Each score is associated to a p-value that describes the probability of the result being a true positive result. Aiming to facilitate the understanding of the results, we have divided the ranking score in four categories.
